# Emergency Department Presentation of a Patient with Altered Mental Status: A Simulation Case for Training Residents and Clinical Clerks

**DOI:** 10.7759/cureus.2578

**Published:** 2018-05-04

**Authors:** Cody Dunne, Andrew W.J. Dunsmore, Jeff Power, Adam Dubrowski

**Affiliations:** 1 Faculty of Medicine, Memorial University of Newfoundland; 2 Emergency Medicine, Pediatrics, Memorial University of Newfoundland

**Keywords:** simulation, simulation based education, altered mental status, toxicology, medical education, emergency medicine, training, metabolic alkalosis, advanced healthcare directives, residents

## Abstract

Emergency physicians frequently are required to perform timely assessments on patients who are unable to provide a comprehensive history due to an altered level of responsiveness. The etiology of their altered mental status (AMS) causes a diagnostic dilemma due to its wide differential diagnosis. Physicians must use a timely combination of collateral history, physical examination skills, and investigations to diagnose the cause of the patient's AMS, as many of the potential etiologies can be life-threatening if not quickly managed.

For this reason, training learners to perform the required actions accurately and effectively proves difficult during real-life emergencies, where an individual's life may be at risk. Simulation-based education (SBE) offers one solution to this challenge. It allows learners to build confidence by dealing with life-threatening conditions in a safe environment and has been shown to be superior to other forms of clinical training.

This scenario explores learners' comfort in some less-practiced, but very important, areas of medicine including obtaining consent for treatment from a substitute decision maker (SDM), explaining various goals of care, and eliciting an advanced care directive from the SDM. Learners and physicians in all fields of medicine must be able to confidently discuss these subjects with patients and their families in order to provide individualized and appropriate management.

In this simulation, learners will have the opportunity to explore an unusual AMS presentation and develop their clinical and communication skills by working as a team to manage the patient.

## Introduction

This technical report describes the case of a middle-aged female who presents with altered mental status (AMS). This is a common scenario in acute care settings where emergency physicians are frequently required to gather critical information about a patient’s presentation in a time-sensitive manner. Since the patient is unable to inform caregivers about her symptoms and medical history, the physician must use ancillary resources such as chart reviews, collaborative history, and information from emergency medical services (EMS) personnel, and may even need to search the patient’s belongings. Obtaining a wealth of information will help the physician narrow the wide differential diagnosis quickly in order to make lifesaving interventions [1].

In this case, the patient has chosen to pursue natural therapies for her advanced stage breast cancer. It is not intended to be a discussion on the ethics or scientific merit of natural therapies. Rather, it is posed to be an introduction for medical residents and clinical clerks (the learners) about the potential use and misuse of these various agents. Many therapies in excess, such as sodium bicarbonate application or colloidal silver use, could lead to a patient presenting with AMS.

Additionally, as the learners discover the patient’s prior wishes from the collaborative history, it presents a management dilemma, as they now must seek direction from the patient’s partner on how to proceed. This explores the learner’s ability to have an open discussion with a patient’s family member about the seriousness of the patient’s presentation and to elicit a decision regarding the patient’s future care while being empathetic with the relative.

This training will be achieved through simulation-based education (SBE), which has been shown to create a safe learning environment for learners where they can make real-life decisions without the potential of adverse patient outcomes. It also has been shown to be superior to other methods of clinical instruction [2].

The overarching goals for the simulation are to provide an introduction to a toxicological assessment, help the learners develop an approach to a patient presenting with AMS, as well as give learners a practical experience in communicating with a patient’s substitute decision-maker in regards to their advanced health-care directives. These goals are formalized in the form of specific learning objectives for senior and junior learners:

Junior learner

1.  Obtain a collaborative patient history. 2. Obtain consent for immediate medical management and an advanced care directive. 3. Interpret an arterial blood gas result.

Senior learner

1.  Complete a primary assessment of a patient with an altered level of consciousness. 2. Order and interpret appropriate investigations for a patient with an altered level of consciousness. 3. Initiate the initial management and disposition planning of a patient with an altered level of consciousness secondary to metabolic alkalosis.

## Technical report

Case

A 57-year-old female is brought to the emergency department in a local tertiary care center after her husband notices that she is not responding appropriately and seems to be more fatigued than normal. On arrival, she is clearly obtunded and has an altered mental status. She is promptly transferred to the main resuscitation bay where the learners and their attending physician are waiting to assess her further.

To describe the simulation experience in detail, the context-inputs-Process-product (CIPP) tool has been utilized for this report [3].

Context

This scenario is designed for two students to participate in the simulation scenario simultaneously: a junior and more senior learner, most suitably a clinical clerk and post-graduate year (PGY) 1–2 resident. The aim is for the pair to not only demonstrate the learning objectives assigned to their level but to also show their ability to work as a team. For the senior learner, they are to direct/guide the junior learner through their tasks and recognize when assistance is required. For the junior learner, they should relay accurate/succinct information back to their senior and know when to ask for help. It is also designed such that if a facilitator wishes to only run the scenario with a senior or a junior learner, this is also possible. In this case, they should only apply the appropriate learning objectives as discussed below.

The scenario was run in a simulation facility at a local university. However, the room is set up to replicate a resuscitation bay in an emergency department (ED) in a local tertiary care hospital. The simulation center has a large area complete with a high-fidelity mannequin that acts as the patient. A one-way mirror allows facilitators to observe the proceedings without taking away from the realism of being present in the room.

Inputs

*Personnel* Three simulated people (SP confederates) were required to conduct the scenario. An SP confederate is a person who portrays a patient, family member, or healthcare provider in order to meet the objectives of the simulation [4]. SP confederates can also participate in providing feedback to the learners so that they receive a patient/colleague's perspective [5]. This option, however, is not utilized in this simulation run.

One confederate portrays a nurse who provides task assistance to the team with actions such as obtaining intravenous (IV) access and providing them with investigation results. The second confederate is the patient’s husband. He is interviewed by the junior learner, collecting information about the patient’s history and providing the team with decisions regarding the patient’s care. The third confederate is the attending physician. This person is largely used as a resource for the learners and to accelerate parts of the simulation that are not the focus of the learning objectives (see below). The facilitator could vary the attending physician’s level of support depending on the level of the two learners.

*Equipment* The learning environment is set up similar to that of a resuscitation bay in the ED, which provides the resources required to complete the simulation successfully. Subsequent runs of the simulation could vary in equipment availability depending on the site. 

This scenario is conducted with the following equipment:

o High fidelity mannequin (note: an SP or low fidelity mannequin could be used here; however, certain aspects of the simulation would have to be modified – for example, placing an IV line) o Cardiac monitor/defibrillator o Intravenous (IV) access supplies (16 or 18 gauge IV catheter) o 0.9% saline in 1L bags o Foley catheter o Glucometer o Intubation/oxygen supplementation supplies o Telephone

**Figure 1:**
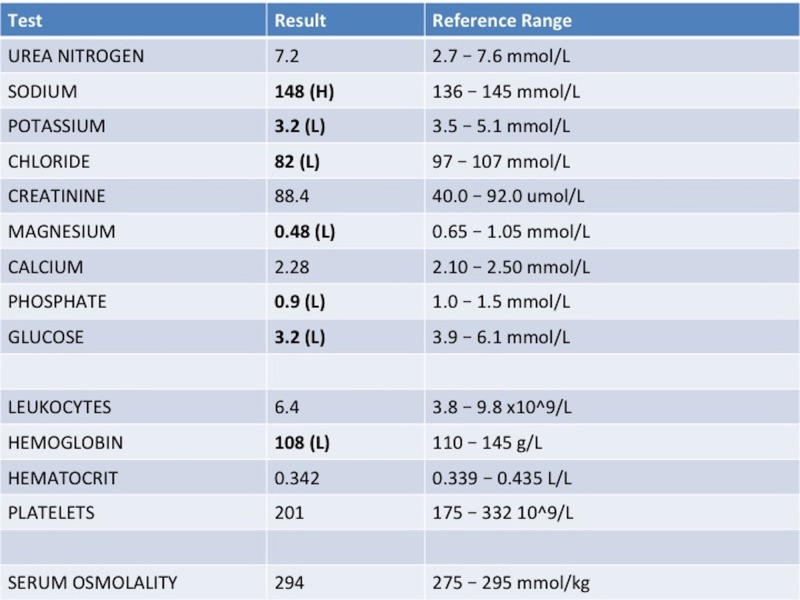
Sample bloodwork for a patient presenting with altered mental status secondary to metabolic alkalosis

**Figure 2:**
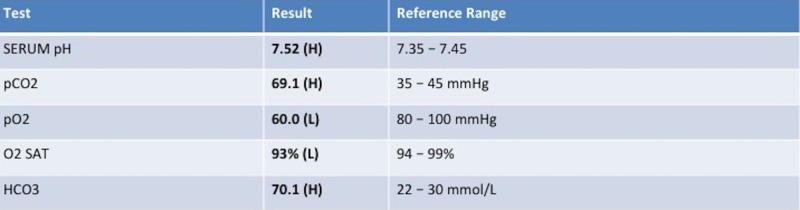
Sample arterial blood gas result for a patient presenting with altered mental status secondary to a metabolic alkalosis

**Figure 3:**
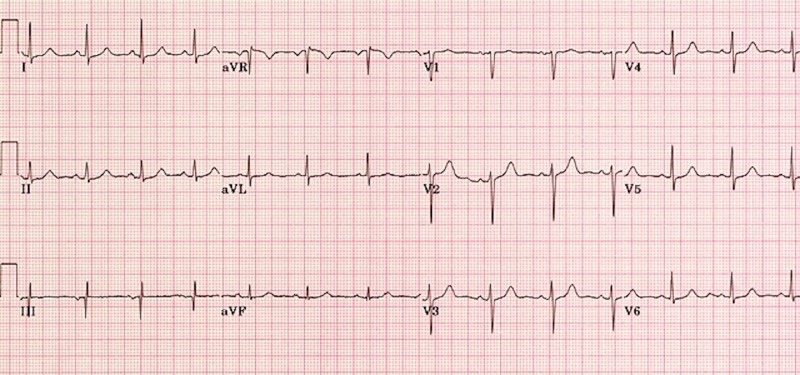
Sample normal electrocardiogram for a patient presenting with altered mental status secondary to metabolic alkalosis

**Figure 4:**
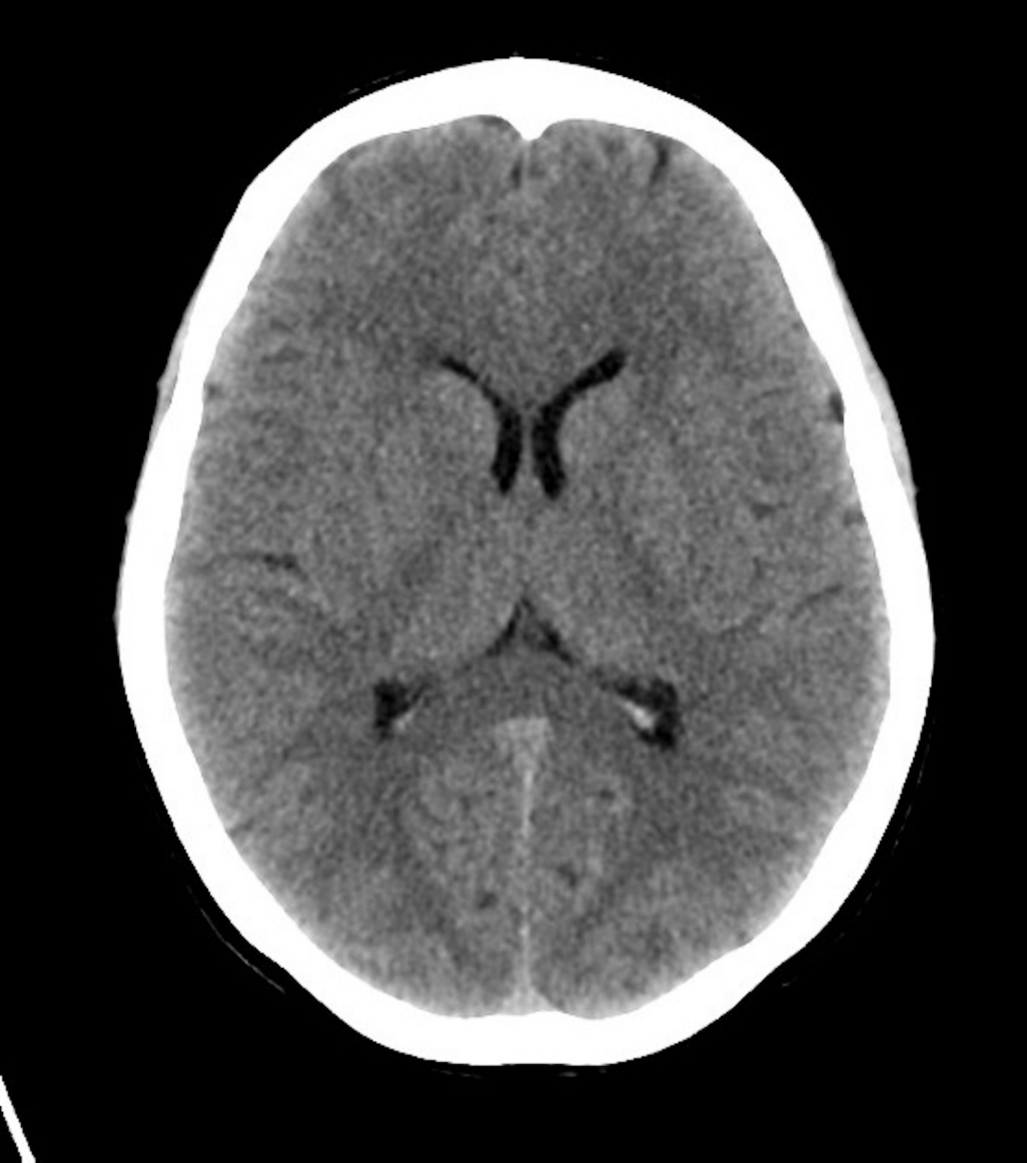
Sample normal computed tomography for a patient presenting with altered mental status secondary to metabolic alkalosis

In addition, Figures 1-4 show various lab results supplied to the learners if they were ordered.

Process

*Pre-briefing* The aim of the pre-briefing is to prepare the learners for the simulation. First, it includes a discussion on the limits of the simulation. The conversation focuses on what the mannequin is and is not able to do, and how some aspects are accelerated (for example, receiving the blood work results) due to time constraints. The “basic assumption,” first discussed by Rudolph et al., is also conveyed to learners at this time [2]. It suggests that all participants are intelligent and that everyone shares a common goal of trying their best and improving from the simulation. The initial run of this scenario is for formative purposes only, and the learners are told about this during the pre-briefing.

The learners are then provided with the pre-scenario before being allowed to enter the simulated ED room.

Pre-scenario

You are completing your rotation in the local emergency department. A 57-year-old female is brought in by her husband when he noticed that she was unusually fatigued. In triage, the nursing staff notices the patient is obtunded so they move the patient to the resuscitation bay with the help of her husband. The attending physician directs the clinical clerk to get collateral information from the husband and directs the resident to begin the initial assessment of the patient. S/he advises that should either of you require assistance, s/he will be nearby.

*Scenario* The scenario begins as the learners approach the patient and husband. The junior learner should introduce themselves and their role to the husband and confirm his identity. They should then begin to take an appropriate medical history from him. Table 1 lists the key points the learner should obtain from the husband.

**Table 1:** A summary of the critical information the learners obtained from the patient’s husband during the medical interview

Category	Information Provided by the Patient's Significant Other
Identification	57-year-old female
Chief Complaint	Increased fatigue
History of Presenting Illness	Found the patient in the bathroom today - drowsy She had been not easy to arouse x1/7 Today, when he found her, she was incoherent when responding No signs of trauma (ex: hitting head) Diagnosed with breast cancer x5/12, stage IV She refused medical interventions, opting instead for natural treatments Has been applying sodium bicarbonate paste three times a day Applies extra thick to the open lesions, especially to her breast Known to ingest paste in recent weeks
Past Medical History	Asthma Menopausal
Medications	Nil
Allergies	Penicillin
Family History	Mother: deceased - age 60 years' old (metastatic breast cancer) Father: deceased - age 76 years' old (myocardial infarction) No siblings No children
Social and toxicology history	No alcohol, tobacco, acetaminophen, or salicylates Two joints of marijuana per day No other illicit drugs Worked as a teacher before diagnosis

Once the clerk believes that they have enough information collected, s/he should return to the senior learner and present it in a logical manner. At this time, if the resident believes the clerk missed pertinent information, they can redirect the clerk back to the husband with some direction on what they were missing. 

Once satisfied with the history, the senior learner should recognize that this individual will require admission and request that the junior learner obtains consent to begin initial management and collect the patient's advanced care directive from the husband. If the clerk is unsure what goals of care could be provided, then the resident may provide clarification on various interventions, such as intensive lifesaving care (ex: cardiopulmonary resuscitation), medical care (ex: IV medications or surgery, if required), or comfort (palliative) care. If the clerk demonstrates an empathetic approach while appropriately explaining the situation to the husband, he selects intensive lifesaving care. If this is not the case, the husband will become more withdrawn and not willing to select an option for the clerk.

After the junior leaner returns to the senior learner, they should present the husband’s response. If the resident is satisfied with the presentation, the nurse confederate prompts the clerk to attempt to interpret the arterial blood gas (ABG) result collected (Figure 2). The clerk can also be prompted as to why the results are as presented, and they should identify the patient's self-application of bicarbonate paste that they learned about from the collateral history.

When the senior learner approaches the patient, the nurse informs them that the patient has been deteriorating since arriving at the ED. The resident should initiate a primary survey by requesting the patient’s present vital signs, assessing the patient's responsiveness (comparing it to the Glasgow Coma Scale), and auscultating the patient's chest for signs of breathing and circulation. All of these findings are summarized below in the learning objectives table.

If the resident doesn't verbally identify their working differential diagnosis at any time, the staff confederate can also prompt for this. A satisfactory answer would include correctly identifying at least four items that could be causing the patient’s condition, including a metabolic disturbance based on what the clerk obtained in the history and their initial physical examination.

The senior learner should also request that the nurse obtains IV access, applies the cardiac monitor, and starts oxygen therapy using nasal prongs aiming to keep the patient’s oxygen saturation (SpO2) greater than 92%. Once these actions are completed, the nurse informs the resident that the patient’s SpO2 cannot be maintained above 85% with the nasal prongs, and the resident should then suggest that endotracheal intubation is required.

The attending physician confederate says s/he will complete the intubation while the resident begins to order investigations. The resident should ask the nurse to initiate the following investigations [6]: o Bloodwork: Complete blood count (CBC), extended electrolytes, glucose, blood urea nitrogen (BUN), creatinine, and serum osmolality o Toxin screening panel, including acetaminophen, ethanol, and salicylate levels o ABG (or venous blood gas, which would be a reasonable substitute) o Computed tomography (CT) of the head o Electrocardiogram (ECG)

Figures 1-4 are presented to the resident to interpret in various time frames. The sample ECG (Figure 3) and computed tomography (CT) (Figure 4) provided are both within normal limits and are used to suggest to the learners that the etiology of the AMS presentation is not cardiac or neurological in origin (specifically secondary to an arrhythmia, a stroke, or a mass lesion). Based on the investigations provided, the resident should suggest that metabolic alkalosis is most likely causing the patient’s current condition. If they don't suggest this on their own, the staff confederate should prompt them to. When asked what their next step in management will be, the resident should suggest giving the patient IV 0.9% saline and contacting the intensive care unit (ICU) for consultation. The telephone is then handed to the senior learner and they should summarize the case to the intensive care physician confederate using the appropriate situation-background-assessment-recommendation (SBAR) format [7-8].

The situation ends as the patient is accepted and transferred to the ICU.

*Learning Objectives* Tables 2-3 outline what is required from each respective learner during the simulation. They provide objective checklists designed for the formative assessment of learners to guide the feedback.

**Table 2:** A summary of learning objectives for the junior learner and associated expected actions SDM = Substitute Decision Maker; ACD = Advanced Care Directive

Expected Action	Findings/Outcome	Completed (Y/N)
Learning Objective #1: Obtain a collaborative patient history		
Introduce self and role		
Obtain patient ID and chief complaint	Refer to Table 1	
Obtain history of presenting illness	Refer to Table 1	
Obtain past medical history	Refer to Table 1	
Obtain medication information	Refer to Table 1	
Obtain allergy information	Refer to Table 1	
Obtain patient's family history	Refer to Table 1	
Obtain social and toxicological history	Refer to Table 1	
Present an accurate and succinct case summary to attending physician	The physician either directs them to collect more of the history or to obtain an advanced care directive	
Learning Objective #2: Obtain consent for immediate medical management and an advanced care directive		
Acknowledge patient’s prior naturopathic wishes		
Obtain informed consent for immediate medical management from SDM	SDM is agreeable to immediate medical investigation and management	
Discuss three different goals of care levels		
Elicits a decision from SDM regarding ACD	SDM selects intensive lifesaving care	
Learning Objective #3: Interpret an arterial blood gas result		
Recognize pH is alkalemic (>7.4)	Physician enquires about whether it is most likely respiratory or metabolic etiology	
Recognize likely metabolic in nature	Physician enquires what is the most likely etiology based on the patient's collaborative history	
Identify exogenous bicarbonate application as likely etiology		

**Table 3:** A summary of learning objectives for the senior learner and associated expected actions LOC = Level of Consciousness; ABCs = Airway-Breathing-Circulation; IV = Intravenous; ABG = Arterial Blood Gas; ECG = Electrocardiogram; CT = Computed Tomography; ICU = Intensive Care Unit

Expected Actions	Findings/Outcome	Completed (Y/N)
Learning Objective #1: Complete a primary assessment of a patient with an altered level of consciousness		
Request initial vital signs	Blood pressure = 134/78, respiratory rate = 8, heart rate = 84, temperature = 36.9, oxygen saturation = 84%, glucose = 4.9	
Assess LOC	Glasgow Coma Scale: 8 (Eye Opening Score = 2 open to painful stimuli; Verbal Score = 2 incomprehensible sounds; Motor Score = 4 withdraws to pain)	
Assess ABC	Airway is patent. Breath and heart sounds are heard over the thorax. Appears to be a decreased breathing effort. The patient is not well perfused (cyanotic extremities and peri-orally)	
Request cardiac monitor and IV access	Nurse completes these tasks	
Initiate oxygen therapy	Patient’s oxygen saturation does not go higher than 86% with nasal prongs	
Initiate intubation procedure	Attending physician offers to complete this while the resident begins ordering the initial investigations	
Learning Objective #2: Order and interpret appropriate investigations for an altered level of consciousness patient		
Order & interpret complete blood count, extended electrolytes, glucose, blood urea nitrogen, creatinine, and serum osmolality	Refer to Figure 1	
Order & interpret toxin screening panel	The nurse reports it is negative for any toxins	
Order & interpret ABG	Refer to Figure 2	
Order & interpret ECG	Refer to Figure 3	
Order & interpret CT (head)	Refer to Figure 4	
Identify most likely etiology	Attending physician agrees and requests what their next step would be	
Learning Objective #3: Initiate the initial management and disposition planning of a patient with a decreased level of consciousness secondary to a metabolic alkalosis		
Order IV isotonic saline in a 1L bolus	No change in patient's status	
Consult ICU/Admitting Service		
Present the case succinctly/accurately to ICU/Admitting Service	Admitting service accepts patient	
Scenario END		

*Debriefing* A debriefing session is conducted immediately following the simulation. The goal of the session is to review the simulation and discuss areas where the learners face obstacles or stray from the expected actions (based on Tables 2-3). Facilitators first review the confidential setting of the debriefing session. The facilitators conduct the session following a debrief model based on the principles of the advocacy-inquiry approach and frame discovery [9-10].

*Post-scenario Didactics* This portion of the simulation is tailored specifically to the group completing it. The goal is to supplement the team’s current knowledge by filling any specific knowledge gaps that they displayed over the course of the simulation. It also was used to solidify prior knowledge.

Product

The expected outcomes for each learner are highlighted by their respective learning objectives:

*Junior Learner* 1.  Obtain a collaborative patient history. 2. Obtain consent for immediate medical management and an advanced care directive. 3. Interpret an arterial blood gas result.

*Senior Learner* 1.  Complete a primary assessment of a patient with an altered level of consciousness. 2. Order and interpret appropriate investigations for a patient with an altered level of consciousness. 3. Initiate the initial management and disposition planning of a patient with an altered level of consciousness secondary to metabolic alkalosis.

## Discussion

This scenario introduces learners to the practical application of many skills developed through medical school and early residency. For the junior learner, the focus is largely on their ability to communicate effectively and empathetically with a person in distress. Medical students often have their first encounter with a patient or family member in distress unexpectedly when they begin clinical rotations. This is a critical time for both the learner and the patient, as what is said and how it is said by the medical profession often sets the tone for the remainder of the person’s healthcare experience [11]. Proper training and exposure through simulation allow junior learners to adequately practice presenting information in an appropriate, considerate manner while still completing their medical tasks and responsibilities. Only through practice can learners find their own style of providing bad news and eliciting critical decisions from patients and caregivers.

For the senior learner, this scenario emphasizes an introduction to the toxicological assessment and assessment of a patient with altered mental status. AMS is frequently encountered in the emergency department and presents a significant large differential diagnosis. By allowing the learner to practice the initial assessments and understand how to order initial investigations, they can begin to develop practices that will better prepare them when faced with a similar patient in an actual emergency scenario.

If training facilities also wish to assess teamwork, this scenario offers the opportunity to incorporate additional learning objectives. The management of the junior learner by the senior learner and the communication between the two can be demonstrated frequently throughout the case. To increase the difficulty of this scenario, facilitators are able to make the attending physician play a less supportive role, which will require the senior resident to take on more of a leadership role.

## Conclusions

This case presents residents and clinical clerks with an opportunity to train with an unusual case of exogenous-induced metabolic alkalosis. However, the objectives assessed throughout the case are core to any learners skill set: communicating clearly and with empathy, assessing a patient with altered mental status, and quickly identifying the need to consult for further assistance.
